# Perceptions of resuscitation care among in-hospital cardiac arrest responders: a qualitative analysis

**DOI:** 10.1186/s12913-020-4990-4

**Published:** 2020-02-27

**Authors:** Samyukta Mullangi, Rohan Bhandari, Porama Thanaporn, Mary Christensen, Steven Kronick, Brahmajee K. Nallamothu

**Affiliations:** 1 Department of Health Policy and Research, Weill Cornell Medicine, 402 East 67th St, LA-202, New York, NY 10021 USA; 2 Department of Internal Medicine, Michigan Medicine, Ann Arbor, MI USA; 3 Institute for Healthcare Policy and Innovation, University of Michigan, Ann Arbor, MI USA; 4 Department of Emergency Medicine, Michigan Medicine, Ann Arbor, MI USA

**Keywords:** Quality improvement, In-hospital cardiac arrest, Resuscitation, Qualitative research, Health manpower, Patient care team

## Abstract

**Background:**

In-hospital cardiac arrests (IHCA) occur commonly and are associated with poor survival and variable outcomes. This study aimed to directly survey IHCA responders to understand their perceptions of resuscitation care.

**Methods:**

As part of a quality improvement initiative, we surveyed participating providers of IHCAs at our institution from Jan 2014 to May 2016. The survey included unstructured free text feedback, which was the focus of this study. We systematically coded the free text and organized identifiable latent themes using thematic analysis.

We used the natural timeline of an IHCA – pre-arrest, arrest, and post-arrest – for organization of the identifiable latent themes, and created a separate category for holistic remarks that arched across the timeline.

**Results:**

We identified 172 IHCAs with a mean of 1.7 responses per arrest (range: 1–8 responses). The mean age of this patient population was 59 years at the time of arrest, and 107 (62%) were men.

We identified several themes - [1] issues around code activation and code status characterized the pre-arrest period [2] ,team interactions and issues around supplies/equipment dominated the intra-arrest period, and [3] code cessation and transitions of care typified the post-arrest period. Holistic remarks focused on attentiveness paid by the arrest team to patient comfort and family. Some comments reflected positive experiences but most focused on areas of improvement consistent with the initiative’s purpose. In certain cases, we identified a tension between the need to balance established resuscitation protocols with flexibility required by real-life circumstances.

**Conclusions:**

Directly surveying those who participated in IHCAs led to novel insights about their experiences. Our findings suggest that parsing through such qualitative feedback can help hospitals identify areas of improvement, modulate expectations, temper emotions, and refine protocols.

## Background

In-hospital cardiac arrest (IHCA) affects approximately 200,000 patients in the United States annually [1]. Quality improvement efforts have largely focused on closely adhering to algorithmic protocols and facilitating first-responder training [2–4]. Despite evidence of modest improvements, efforts to successfully resuscitate patients remain challenging and outcomes continue to be poor, with approximately 20% of patients surviving, on average, to discharge [5]. Furthermore, recent evidence suggests wide variation in quality of resuscitation efforts leads to potential differences in survival-to-discharge rates and neurologic outcomes [6, 7].

An important contributor to the variability in outcomes may be the unique nature of cardiac arrest teams across hospitals. For example, respiratory therapists, social workers, pharmacists, nursing personnel and physicians all play different roles in a resuscitation in spite of common background training in Advanced Cardiac Life Support (ACLS) [8] and the recognition for inter-professional collaboration [9]. To our knowledge, no previous study has systematically asked a broad range of IHCA first-responders across multiple disciplines about their experiences with resuscitation care to directly capture their perceptions on performance. This is an important gap for two reasons. First, perception drives individual behavior and institutional culture, and nearly all process improvement strategies, from standardized reporting to guideline implementation, rely in part on understanding and managing culture [10]. Second, IHCA is a complex clinical process that involves team members who often do not know each other prior to arrival at a cardiac arrest [11, 12]. Under such emergency circumstances, success does not rely so much on the performance of an individual provider as it does the overall interactions of multiple providers and the system [13]. Information on the perceptions of global operations of a resuscitation team could lead to novel insights.

Qualitative analysis is a methodological tool ideally suited to evaluate perceptions of first-responders after IHCA. It allows us to examine both the technical and procedural aspects of resuscitation from diverse informants while unpacking complicated issues like care coordination. Further, it can complement earlier quantitative methods in this area by generating new hypotheses and explanatory models for future investigation [14]. Our hope in conducting this study was that by understanding themes that arose from perceptions using qualitative analysis we could develop new areas around topics identified by first-line responders to provide a framework to guide improvements in resuscitation care.

## Methods

### Data sources and study cohort

This study is based on a quality improvement initiative to improve resuscitation care at our institution, a tertiary hospital that averages 2 million clinic visits and 50,000 discharges yearly. IHCA teams are interdisciplinary and comprise a rotating crew of participants. Responses to IHCA are led by house staff from the Department of Internal Medicine with attending support and supervision. Additionally, teams also form ad-hoc to attend to rapid-responses, which represent an early response system for impending cardiac events.

For the past 5 years, as part of an internal quality improvement effort, we have been collecting and centralizing survey feedback from IHCA first-responders in an anonymous manner to encourage frank reporting and explore a virtual “de-brief” tool. The survey, sent out to staff listed in opened code-narrators (the electronic tool embedded in our EPIC-based electronic health record system to document the IHCA) within 1–2 weeks of an arrest, includes a series of structured and open-ended free-text responses that are meant to gauge the perceived quality of each IHCA event. Responding to the survey was not mandatory, and there were no reminder emails to nudge participation. Responses are collected and then maintained in a confidential database where they are routinely reviewed by hospital leadership in IHCA and resuscitation care. Responders included resident physicians, fellows, attending physicians, nurses, respiratory therapists, and pharmacists. We used data for IHCA events from Jan 2014 to May 2016 for this study. This study was given an exemption by the University of Michigan Institutional Review Board, and the requirement for informed consent was waived.

Over the study period yielded, we included 172 IHCA events regardless of the presenting cardiac rhythm as long as the resuscitation team was activated and provided care and at least one completed survey response was available. We included data on multiple cardiac arrests for the same individual patient (*n* = 4). We did not include cardiac arrests that occurred in operating rooms, the cardiac catheterization lab, and the emergency department, as these arrests are structurally different at our institution and do not routinely involve the resuscitation team. We did, however, include arrests that occurred in the medical procedural unit. The number of responses ranged from 1 to 8. We entered all data into MAXQDA, a VERBI GmbH product, to facilitate data review, analysis, and reporting.

### Analysis

Though the mechanism of collecting feedback was dissemination of an online survey tool, the data analyzed for this qualitative analysis was the free-text portion of the feedback. We chose thematic analysis as our approach to evaluate the dataset, as it is well suited for investigating complex interpersonal interactions, especially for perceptions around emotionally charged events like IHCA. Thematic analysis enabled us to analyze these data sources in terms of the principal concepts or themes with the ability to provide supporting quotes that point toward these concepts. Our goal was to seek out and conceptualize the latent themes through an examination of patterns and structures within the open-ended data using a recursive process given a lack of a prior established framework in this area [15]. Initially, we took an inductive approach to generate codes from a subset of the data (*n* = 25) before we implemented a consistent and comprehensive coding system for the entire dataset. The process of code development and assignment were conducted by two members of our team (SM, RB) working together with additional team members (SK, BKN) to promote more in-depth discussion and understanding of the conceptual content of the data. Findings were then reviewed with full team members. Finally, we examined the codebook to identify broader themes (recurrent/unifying ideas that described participants’ experiences).

The authors had full access to the data and take full responsibility for its integrity. All authors have read and agree to the manuscript as written.

## Results

We analyzed 172 arrests that met our specific parameters during this 2.5-year period. The mean age of this patient population was 59 years at the time of arrest. One hundred seven of the 172 patients were male (62%). Sixty-eight arrests occurred on the floor, 92 in the ICUs, and 20 in procedural or imaging suites.

We used the natural timeline of an IHCA – pre-arrest, arrest, and post-arrest – for organization of the identifiable latent themes, and created a separate category for holistic remarks that arched across the timeline.

### Key themes characterizing experience with IHCA

Sample quotations for each of these themes have been included in Table 1.

**Table 1:** Sample quotations for the compiled themes

Timeline of arrest	Themes	Sample Quotations
Pre-Arrest	Code Activation	(A) While an ICU bed was being prepared, the patient ended up coding on the floor. (B) This arrest happened close to shift change in an ICU setting. The ICU staff required assistance from the floor code teams.
	Code Status Issues	(C) The patient was DNI but not DNR making resuscitation efforts difficult. (D) When the patient lost BP (blood pressure), MD confirmed with the wife we weren’t doing chest compressions per his code status (he was intubation only). (E) Alert the family sooner that death of the patient is imminent and attempt to change the code status from full to DNR (do not resuscitate). The patient was reintubated for the second time on the unit in less than 12 h.
Arrest	Team Interactions	(F) We identified the loss of pulse immediately, started CPR (cardiopulmonary resuscitation) while the patient was in the chair, and delivered the first shock before the code team arrived and the patient regained consciousness. (G) The MD in charge did a wonderful job, very clear with his directions, wore the yellow sign. He came back to our unit to assure that we didn’t need anything else after the code. (H) The team leader was not initially evident. Orders were coming from many different people. (I) The code ran very smoothly. The only complaint I heard was that there were too many people in the room. Some of the ICU nursing staff suggested that [these people] leave the room.
	Supplies and Equipment	(J) The supplies on the cart were quickly used up. (K) …The drugs that anesthesia usually brings, such as phenylephrine, were not available.
Post-Arrest	Code Cessation	(L) The length of time and amount of medication we used in this code was unacceptable. I strongly feel that this code should not have lasted as long as it did. (M) The team’s ability to “let go” was an issue. The patient was gone at least an hour before we stopped coding her.
	Transitions of Care	(N) Floor nurses need to have their code documentation done when or shortly after the patient transfers to an ICU. So if the patient arrests again (like this patient did), the ICU can start and document the code in real time instead of putting together the variables later. (O) The ICU fellow was called, but did not enter the room to assess the situation. She should have found me as the team leader before deciding not to accept the patient, especially since this patient had been in the unit 4 days prior.
Holistic	Attentiveness to Patient Comfort	(P) Dr. X cannulated this patient for ECMO (extracorporeal membrane oxygenation) during CPR…It was very upsetting. (Q) Unsure as to why the RN placed intraosseous access when he had two functioning lines. The patient was awake at the time it was placed, and found it to be very painful.
	Family	(R) We had taken the step to involve ethics as the patient had a terminal illness, and yet the wife (who did not fully understand the code process and would hang up when discussions were initiated) wanted everything done. (S) During the code, a family member barged in the room, hysterical, and demanded that we stop. The entire team actually stopped compressions and intubation. As the code leader, I asked them to resume CPR, which they did. The family member was not the DPOA.

#### Pre-arrest

First-responders noted different issues that characterized the typical timeline of code activation. To start, responders commented on challenges posed by the geography of the patient in relation to the arrest team, and the time of day at which arrests occurred, some of these around unavoidable aspects like shift change (See Quotes A and B).

Issues around code status also were raised. In one setting, there was incongruence described between complicated orders for nuanced code status in order to respect patient preference (e.g., opting for defibrillation, but not intubation) (See Quote C). In another, a first-responder pointed out the challenges for the primary team to revisit code status with the patient and family (See Quotes D and E).

#### Arrest

Comments classified as relating to code performance generally fell into two broad categories – issues around team interactions (including leadership) and issues around supplies and equipment.

Responders expressed both positive comments and frustration around role identification as well as the processes and responsibilities associated with different posts (see Quote F). Consistent with previous studies, attention was frequently directed toward the team leader, who would either be singled out for praise in the case of a smoothly run code, or targeted for ire if events beyond her (or his) control occurred (see Quotes G and H) [12]. Though the literature around communication has tended to focus on the intra-code procedures, such as closed-loop communication, our first-responders highlighted even peri-code communication as an issue worth addressing.

Finally, crowd control was a frequent concern even when codes were positively viewed (see Quote I). The presence of the inactive audience in the room was usually felt to be a hindrance, and the responsibility of crowd control was laid at the feet of the team leader as one of his or her responsibilities.

Perceptions of code performance were also dependent upon availability of supplies and equipment. Interestingly, first-responders described in great detail the challenges that supplies and equipment imposed upon the running of a successful code even when several of those supplies were not necessarily linked to ACLS (see Quotes J and K).

#### Post-arrest

Central to the perceived success of an IHCA was a smooth termination, adequate disposition, and timely and accurate documentation. The decision of when to call for termination was frequently mentioned as challenging (see Quotes L and M). It was felt to be largely related to the team’s ability to ‘let go’ and recognize that further resuscitation efforts were futile.

Disposition plans for immediate survivors and the importance of documentation, highlighting both accuracy and timeliness, were commonly mentioned (see Quote N). Floor teams that were able to upload their documentation of the arrest to the chart in a timely fashion for the ICU teams were praised. Additionally, the importance of peri-code communication was also highlighted to ensure safe transitions of care (see Quote O).

#### Holistic

Holistic remarks that did not fit neatly into a specific arrest timeline were largely about the team’s perceived attentiveness to patient and family comfort. Despite the recognition that few things about a cardiac arrest and subsequent resuscitation can be comfortable, team members remained on the lookout for small opportunities to make the experience more tolerable for the patients (see Quotes P and Q). Responders also highlighted that family members did not always seem to understand what a code status is and that this we viewed as an opportunity for improvement (see Quote R). One responder also pointed the tension that occurs when family members who are not legal decision-makers have strong opinions about the choice to pursue resuscitation (or vice versa) (see Quote S).

## Discussion

This study aimed to capitalize on survey data obtained from an internal quality improvement initiative to better understand the experiences of first-responders reacting to IHCAs. We uncovered several themes, spanning environmental, operational, and cultural topics, that characterized the perceptions of first-responders to resuscitation care. Importantly, we did not examine actual quality of the resuscitation as others (including ourselves) have previously done by assessing processes or outcomes in quantitative studies [7, 16–18]. Instead, this study highlighted several factors about the complexities of an IHCA directly based on perceptions. These included: variability in patient characteristics and circumstances, a broad range of knowledge among staff, differing challenges across first-responders, the need for professionals to adapt processes quickly to a dynamic setting, and the importance of engaging family.

Consistent with previous literature in this field, our study suggests that first-responder perceptions hold potentially valuable information for broad application to process and care quality improvement targets [19]. For example, one key discovery we uncovered was the need for balancing established protocols with the flexibility to deviate from these protocols in response to real-life challenges. In Table 2, we list some of the myriad expectations that first responders hold of the “ideal” IHCA, as informed by their ACLS training. At times, tension between these challenges and the expectations that first-responders had for an “ideal” code that is often taught in ACLS and other educational interventions can be real and we have now tried to incorporate this teaching during our monthly review with staff on the resuscitation teams.

**Table 2:** Expectations held by first-responders

Patients will have easily accessible and congruent code statuses	
Impending clinical decline has been recognized by primary team	
An authoritative team leader will assume responsibility and assign roles	
There will be no shortage of supplies and all equipment will function well	
All team members will be familiar with protocols	
The code will be properly terminated in a timely fashion	
Documentation will be accurate and quick	
Disposition of the patient will be safe	
Family will be respectfully debriefed and attended to	

Collectively, our results recognize real-world challenges that require quick adaptation to complex situations. For example, most hospitals (including our own) operate emergency response teams that are formed ad hoc and rapidly disintegrated, with compositions always in flux [20]. We have realized that to aim for excellence requires us to anticipate potential failures in the system and organize to mitigate it. This is what distinguishes HROs, but such flexibility is not easily captured in available quality metrics [8, 21]. Though there exists good data backing clinical targets such as time-to-compression, time-to-defibrillation and risk-standardized survival rates to discharge – areas in which our hospital has consistently performed at the top of the national American Heart Association Get-With-The-Guidelines-Resuscitation registry – there are not similar metrics for creativity, leadership, teamwork, and critical thinking. Many of these essential issues were raised in the text we reviewed. As a result of this study, we have shifted our thinking to better understand how these important variables may be more effectively measured.

Our dataset had several limitations. First, it originated as the byproduct of an internal quality improvement initiative, and so its purpose was not for academic study but rather to serve as a starting point for systematic data collection about high-frequency critical events. Because the survey was anonymous, we did not have information on the specific role of many of the responders (e.g., nurse, physicians) or the details of the arrest to review. Thus, we could not confirm criticisms with other data sources to show evidence of actual harm. Additionally, anonymity that de-linked feedback from clinical encounters also made it impossible to go back and complete subgroup analyses related to patient outcomes. Although we still feel there is value in understanding perceptions, it is important to note that perceptions could contradict each other at times or even describe care practices that may not be optimal. For example, if a resident physician described an inadequate supply of calcium in the chart, then this may indicate a lack of knowledge regarding the drug’s use during IHCA as much as it reflects a gap in quality. Similarly, if a team member highlights a certain delay that is not substantiated in documented time periods, perhaps that speaks to discomfort with managing a critically ill patient. In both cases, the value is in understanding the concerns raised by first-responders during these stressful events that could be used to improve the system.

Second, completion of the survey was not binding, and there were no reminder emails to nudge completion, so response rates were variable, and may have been biased toward IHCAs where major events arose. Poorer outcomes engendered the most responses, which skews interpretability of general themes. Finally, this represents the experience of a single medical center and its providers. Our hospital has participated in a national registry and quality improvement initiative for several years, excelling in outcomes that we routinely track and benchmark against other facilities. Even with such a deep investment in this space, we undertook this project because we continue to understand that ongoing quality improvement efforts are required to maintain high performance in a condition like IHCA. Other hospitals are likely to report different perceptions based on the organization of their resuscitation teams as well as their patient populations and the makeup of their providers.

Despite these limitations, however, these data demonstrate how valuable feedback from first-responders can be [22–24]. For example, many hospitals currently struggle to perform debriefing after IHCA given the need for first-responders to return to their primary clinical duties soon after the event. This project showcased the appetite within our staff to reflect on their experiences and enrich each other’s awareness of blind spots and preventable errors. It also showed how an electronic system might serve as an efficient method for soliciting this input. Indeed, a recent survey of best practices associated with survival after IHCA showed that hospitals in the top quintile were likely to conduct immediate debriefing after an acute resuscitation despite the fact that few utilized it [25].

Additional benefits are also possible. IHCAs are often emotionally charged events. Reflecting on and sharing experiences may be beneficial for responders. Previous literature that sought bystanders’ perceptions of out-of-hospital cardiac arrest demonstrated benefit in both identifying knowledge deficits of basic life support in the community, and in helping these witnesses cope and reflect on their emotions after a stressful experience [26]. Finally, in parsing through the comments, it was abundantly clear how differently physicians, nurses, respiratory therapists, and pharmacists, among others, view their roles and contributions in the context of their relationships with each other. The process of giving and receiving feedback can help responders understand each other’s experiences and develop an expanded appreciation of different participants’ contributions [27]. We are exploring use of these comments to help cross-train multiple providers and to help modulate expectations, and refine or troubleshoot protocols.

## Conclusions

Directly surveying those who participated in IHCAs led to novel insights about their experiences. Our findings suggest that parsing through such qualitative feedback can help hospitals identify areas of improvement, modulate expectations, temper emotions, and refine protocols.

## Supplementary information

**Additional file 1.** Spreadsheet with sample quotations for compiled themes.

**Additional file 2.** Copy of survey sent to cardiac arrest first-responders.

## Data Availability

A copy of the original survey, and sample survey responses are included in this published article and its supplementary information files. The full dataset generated during the current study is not publicly available due to privacy concerns and sensitive clinical information that were collected as part of an internal Quality Improvement initiative, but can be made available from the corresponding author on reasonable request.

## Abbreviations

ACLS: Advanced cardiac life support
BP: Blood pressure
CPR: Cardiopulmonary resuscitation
DNI: Do-not-intubate
DNR: Do-not-resuscitate
DPOA: Durable power of attorney
ECMO: Extracorporeal membrane oxygenation
ICU: Intensive care unit
IHCA: In-hospital cardiac arrest
