# Supplementary material for: Perceptions of resuscitation care among in-hospital cardiac arrest responders: a qualitative analysis
Source: BMC Health Serv Res. 2020 Feb 27;20:145. doi: 10.1186/s12913-020-4990-4 (PMC7045452; doi:10.1186/s12913-020-4990-4)
Supplement: Supplementary file 2 — Additional file 2. Copy of survey sent to cardiac arrest first-responders. [file 12913_2020_4990_MOESM2_ESM.pdf]

Please place the arrest patient's event information here (this is in the e-mail message sent to you).

hello

Please describe one issue that could be improved upon within this arrest.

hello

Please answer Yes or No to the following questions regarding this arrest.

	Yes	No
Was the defibrillator used in the AED Mode?	○	○
Were there any problems with Airway management?	○	○
Were there any problems with medication administration or availability?	○	○
Where there any problems with coordination of team efforts by physician in charge?	○	○
Did the Team Leader wear the yellow Team Leader Sign?	○	○
Were there any problems with Presence of necessary unit support staff?	○	○
Were there any problems with availability of supplies in the crash cart?	○	○
Were there any problems with equipment function?	○	○
Were there any problems with ability to transfer the patient, if necessary, to a higher level of care (e.g., ICU or telemetry bed)?	○	○
Was Social Work able to respond?	○	○
If so, was their presence helpful?	○	○

Any other comments about this arrest?

hello

Approximately how long did you remain at the patient's bedside to respond to this call?

hello

If you would like someone to contact you regarding any concerns you may have, please leave your unique name here.

(Optional)

hello

>>
